# Enhancement of Polyacrylate Antiscalant Activity during Gypsum Deposit Formation with the Pretreatment of Aqueous Solutions with Spruce Wood Shavings

**DOI:** 10.3390/ma16196516

**Published:** 2023-09-30

**Authors:** Maria Trukhina, Konstantin Popov, Maxim Oshchepkov, Sergey Tkachenko, Alina Vorob’eva, Olga Guseva

**Affiliations:** 1 JSC “Fine Chemicals R&D Centre”, Krasnobogatyrskaya Str. 42, b1, 107258 Moscow, Russia; truheniy-mv@yandex.ru (M.T.); maxim.os@mail.ru (M.O.); viri2709@yandex.ru (A.V.); guseva@travers.su (O.G.); 2 Department of Chemical and Pharmaceutical Technologies and Biomedical Pharmaceuticals, Mendeleev University of Chemical Technology of Russia, Miusskaya sq. 9, 125047 Moscow, Russia; s.tkach.8@gmail.com

**Keywords:** wood shavings, gypsum, scale inhibition, polyacrylate

## Abstract

Considerable efforts are made worldwide to reduce inorganic scale in reverse osmosis plants, boilers and heat exchangers, evaporators, industrial water systems, geothermal power plants and oilfield applications. These include the development of new environmentally friendly antiscalants and the improvement of conventional ones. The present report is dedicated to the unconventional application of spruce wood shavings in combination with polyacrylate (PAA-F1) in a model case of gypsum scale formation. The electrical conductivity of freshly prepared gypsum solutions with a saturation SI = 2.3 and a concentration of 0.05 mol·dm^−3^ was analyzed over time at 25°C. It is demonstrated that the small amounts of wood shavings (0.1% by mass) alone, after being in contact with CaCl_2_ and Na_2_SO_4_ stock solutions for 15 min, increase the induction time t_ind_ by 25 min relative to the blank experiment (t_ind_^blank^). In the presence of PAA-F1 (0.1 mg·dm^−3^), the difference Δt_ind_ = t_ind_ − t_ind_^blank^ constitutes 110 min, whereas the sequential treatment of the stock solutions with the shavings followed by PAA-F1 injection gives Δt_ind_ = 205 min. The observed synergism is associated with the selective removal of colloidal Fe(OH)_3_^solid^ and Al(OH)_3_^solid^ nanoimpurities from the stock solutions via their sorption to the well-developed surface of wood. Wood shavings therefore represent a very promising and environmentally friendly material that can significantly improve the effectiveness of conventional antiscalants.

## 1. Introduction

Scale is a layer of solids that form on the surfaces of industrial equipment through a phenomenon called scaling. During this process, certain inorganic materials (such as calcite, aragonite, gypsum, barite, and calcium oxalate) that were initially dissolved in aqueous phase are deposited under certain conditions on a surface such as pipe walls or heat transfer surfaces. The term “scale” commonly defines a dense crystalline deposit adhering to the surface [1]. Such deposits cause serious industrial problems, including reduced thermal efficiency of heat exchangers, increased pressure drop in pipework, flow oscillations and flow blockage. The estimated cost of fouling in some industrialized countries is approximately 0.25% of their Gross National Product (GNP) [2]. Mitigation of scale deposition on heat exchanger surfaces could be achieved by using chemical additives [1], heat exchanger surface modification, modification of heat exchanger operating parameters [2], and other non-chemical methods, such as the application of permanent magnets and electric fields, the introduction of natural fiber into the fouling liquids, etc. [3]. Among these, the most effective and widely used approach is the application of chemicals that inhibit scale formation at sub-stoichiometric levels [1,4,5,6]. 

However, the universal solution to scale mitigation is hardly possible due to the very complicated and diverse nature of scale formation processes. The development of mineral scale on the surface of industrial equipment involves at least four stages: initiation, transport, deposition, and ageing [1]. The initiation step in the bulk aqueous medium includes, in turn: solid phase nucleation, nucleus growth and aggregation, followed by sedimentation. Each of these stages is affected by different antiscalants to varying degrees. As a result, scale inhibitors extend the cleaning intervals of industrial equipment. Meanwhile, the use of the most common antiscalants (phosphonates, polyacrylates) raises environmental concerns about the discharge of antiscalant-containing sediments [2]. This problem is partly addressed by biodegradable antiscalants such as polyaspartates or polyepoxysuccinates [7]. At the same time, although biodegradable antiscalants slow inorganic fouling, they can increase the potential for biofouling, which is considered to be a very difficult problem to manage [8]. Therefore, the search for new ways to reduce scale in the most environmentally acceptable way [9,10] is more important than ever. Among these, the various combinations of non-chemical methods with antiscalant applications [11,12] seem promising. In this respect, the results of Middis [13] and Kazi [2,14,15] are quite interesting. It has been reported that the addition of small quantities of wood fiber to a calcium sulfate solution can lead to a considerable decrease in the rate of deposit growth. The wood pulp fibers, added to the fouling solution, reduced both the fouling rate and the duration of fouling. Fouling was suppressed for 45 days using 0.25% fiber in the suspension, and the heat transfer rate was found to be constant over the entire period. The mean diameter and length of fibers were 30 mm and 2.5 mm, respectively. In particular, the crystal size and gypsum crystal growth phenomena change in the presence of fibers in a manner similar to polymer additives. Meanwhile, the inflexible microfibers do not seem to act in the same way as the flexible polymers with a molecular mass of 2000 to 4000 Da and a molecular length of around 12 nm [16]. Unfortunately, the lack of important experimental details in [14,15], such as the sorption of calcium ions and particulate matter, does not allow the mechanism of action to be clarified. At the same time, the proposed interpretation of the data (isolation of the heat transfer surface from the deposit and additional turbulence near the tube walls) [2,11] does not seem convincing. Therefore, the main objective of the present study is to test the antiscaling activity of wood fiber, excluding “fiber–heat transfer surface” interactions, e.g., in supersaturated bulk gypsum solutions with an emphasis on the role of natural particulate matter. Gypsum scale formation in artificially prepared supersaturated aqueous solutions was chosen as the model process following the previous publications on wood pulp fiber activity studies [2,14,15]. These experiments were carried out in comparison with a conventional polyacrylate antiscalant (PAA-F1) [16]. 

On the other hand, regardless of the long-term and successive use of antiscalants, both the mechanisms of scale nucleation and growth [17,18,19,20,21,22,23,24] and the mechanism of scale inhibition provided by antiscalants [1,25,26,27,28,29,30,31] remain a matter of discussion. Indeed, some authors adhere to the hypothesis of spontaneous homogeneous nucleation in the supersaturated bulk solution of sparingly soluble salts [19,32], others insist on heterogeneous nucleation on solid nanoimpurities in the bulk solutions [21,23,24], some admit the possibility of homogeneous nucleation at high supersaturations and a heterogeneous one at low supersaturations [17,18], some give priority to heterogeneous nucleation on the heated surfaces of industrial equipment [22], and others emphasize the role of the pre-nucleation cluster homogeneous pathway [20]. As long as the nucleation step cannot be reliably detected and characterized by direct experiments, all of these mechanisms remain hypothetical and can only be confirmed indirectly.

Similarly, there is a wide variety of views on the activity of antiscalants [1,4,5,6,7,25,26,27,28,29,30,31,32]. The scale inhibitors may act in a bulk aqueous medium through several possible routes. They can (i) alter the chemical potential of the precipitating salt by masking cations such as Ca^2+^ and Ba^2+^; (ii) be adsorbed onto sparingly soluble salt embryos, preventing their passage through the critical size barrier; (iii) impart a significant electrostatic charge to the scale nuclei, slowing their aggregation due to electrostatic repulsion between sparingly soluble salt colloids; and (iv) be adsorbed onto growing crystals, inhibiting their further crystal growth. An effective inhibitor may act by more than one of the above routes. However, a critical analysis of these routes reveals numerous gaps in these theoretical approaches, which do not provide a reasonable explanation for a large number of experimental observations [31]. Therefore, our group has recently proposed the hypothesis that an antiscalant does not isolate the sparingly soluble salt embryos or growing crystal surface but likely blocks the natural solid nanoimpurities in the feed water. These impurities then lose their ability to act as nucleation centers, and the scale formation is slowed. If our hypothesis is correct, then the wood macro-fibers can act as a kind of trap for natural nano-impurities, reducing the number of nucleation centers in the bulk aqueous medium. Therefore, the secondary purpose of this work was to test this idea as far as possible. Hopefully, a better understanding of the mechanisms that reduce the accumulation of gypsum deposits in the presence of spruce wood shavings will provide the possibility to downsize or even eliminate the problem in particular situations.

## 2. Materials and Methods

### 2.1. Materials 

Na_2_SO_4_ (GOST 21458-75, RU; 98.94%) and in-house deionized water (Laboratory Reagent Water Type I, ASTM D1193-06 (2011), 0.056 μS/cm) were used in the current study for the preparation of stock solutions. The prepared water passed through filtration (450 nm (Hydrophilic PTFE Millipore Millex-LG Membrane, Merck KGa, Darmstadt, Germany), followed by particle counter and laser dynamic light scattering (DLS) technique measurements of the natural solid impurity content before use (see Table 1). The data indicate that the in-house deionized water satisfies all international standards and maintains the natural level of foreign particles, attributed to CaCl_2_ and Na_2_SO_4_. To obtain the CaCl_2_ solution, ultrapure CaCO_3_ (99%, EKOS-1 Co., Moscow, Russia) was dissolved in ultrapure 36% HCl (EKOS-1 Co., Moscow, Russia) and was later diluted with in-house deionized water. The 20% HCl was slowly poured into an aqueous suspension of CaCO_3_, which was taken in a 10% molar excess over the acid. The resulting mixture was heated to 120°C while being stirred for 2 h to eliminate CO_2_. The reaction mass was cooled and left overnight. The solid residue was removed via titration, and the liquid phase was analyzed to determine its calcium content, which was then diluted to create a 0.1 mol·dm^−3^ stock solution of CaCl_2_. This method has been found to lower the concentration of particulate matter impurities (particles larger than 200 nm) when compared to the commercial CaCl_2_ sample used in our previous study [23], as shown in Table 1. Additionally, the approach results in fewer iron impurities. 

**Figure 1:**
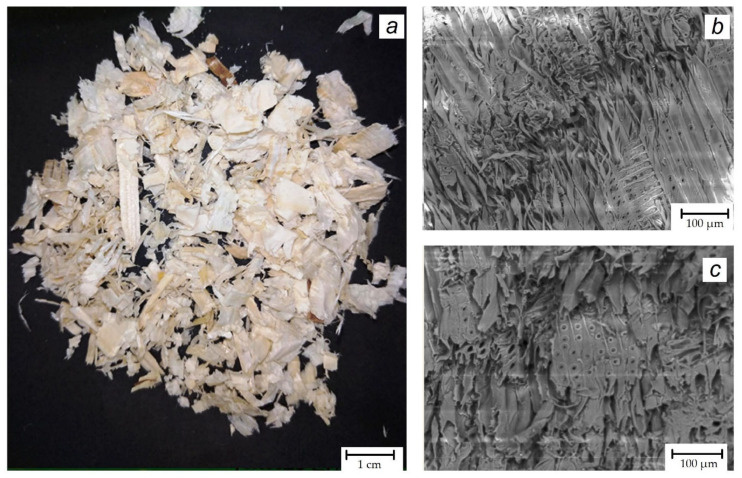
General optical (**a**) and SEM (**b**,**c**) images of spruce wood shavings, used in the present study.

The commercial sample of spruce wood shavings (Figure 1a), which was manufactured as pet litter by Rodnye Mesta Co., Mamadysh City, Russia, was utilized in its original state, except for a gentle rinse with deionized water to remove any occasional dust. The average dimensions of the shavings were 1 cm in length and 0.5 cm in width, with a thickness ranging from 0.01 to 0.1 cm. Nonetheless, the length of certain pieces exceeded 3 cm. Figure 1b,c depict that all shavings had a very rough and well-developed surface. Energy-dispersive X-ray spectroscopy data, processed with QUANTAX 70, Hitachi-High-technologies Corporation, Tokyo, Japan, indicated that typical wood elements O, C and N, are dominant.

**Figure 2:**
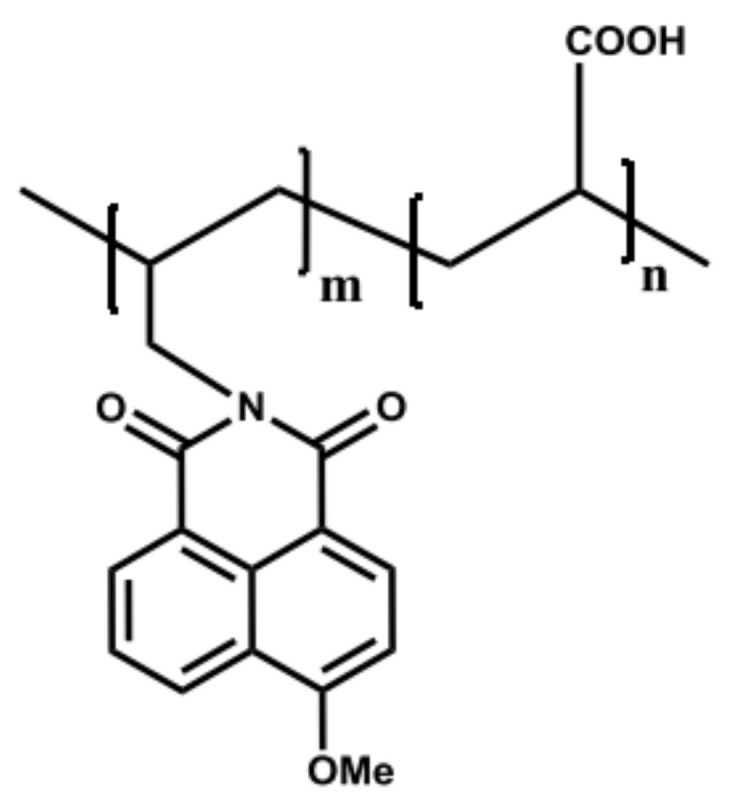
Molecular structure of naphthalimide-tagged polyacrylate PAA-F1.

A co-polymer of N-allyl-4-methoxy-1,8-naphtalimide and acrylic acid (PAA-F1) was synthesized based on the methodology provided in [33]. PAA-F1 is a 5% sodium acrylate polymer with a molecular mass of approximately 4000 Da and a pH value of 5.0. The fluorophore fragment represents 0.5% of the total mass of the PAA-F1 molecule, as shown in Figure 2. It was observed that PAA-F1 functions as an effective antiscalant for gypsum [16] and is superior to PAA due to its ability to be monitored by fluorimetry for concentration measurement.

### 2.2. Scale Formation Procedure

The experiments were performed in a certified laboratory room (ISO 14644-1, class 8) with a limited dust microparticle air phase content, monitored using the Particle Measuring Systems Inc., Boulder, USA, air particle counter (analytical channels 0.3; 0.5; 5.0 µm). The influence of spruce wood shavings on gypsum nucleation was studied by measuring the specific conductivity in batch experiments, conducted at 25 °C. The 0.1 mol·dm^−3^ CaCl_2_ and Na2SO4 stock solutions were prepared using deionized water (see Table 1). They were then subjected to a standard filtration procedure (450 nm; hydrophilic PTFE Millipore Millex–LG membrane, Merck KGa, Darmstadt, Germany) and used in the blank experiment. Then, 50 mL of 0.1 mol·dm^−3^ CaCl_2_ solution was mixed with 50 mL of 0.1 mol·dm^−3^ Na_2_SO_4_ (blank experiment) in a thermostatically controlled cell for conductivity measurements with magnet stirring at 300 rpm. The initial gypsum concentration was 0.05 mol·dm^−3^ in 0.1 mol·dm^−3^ NaCl. As the gypsum solubility in 0.1 mol·dm^−3^ NaCl at 25 °C corresponds to 0.023 mol·dm^−3^ [34,35], the supersaturation index (SI) was 2.3 for all runs. In the present work, SI is defined as SI = (initial gypsum concentration, mol·dm^−3^)/(gypsum solubility, mol·dm^−3^) at 25 °C. Induction times (t_ind_) were measured from the specific conductivity æ [36,37], as the point of deviation of the curve from the plateau. 

The experiment with PAA-F1 was carried out in the same way as the blank one. The antiscalant was added to the Na_2_SO_4_ solution, equilibrated for 30 min, and then both calcium and sulfate solutions were mixed in the cell for conductivity measurements. The PAA-F1 concentration in the Na_2_SO_4_ solution was 0.2 mg·dm^−3^, while in the gypsum supersaturated solution it was 0.1 mg·dm^−3^.

The studies of the effect of spruce wood shavings on gypsum deposition excluded their presence in the scale forming medium. This is the major difference with previous wood fiber experiments reported in [2,13,14,15]. The shavings (0.05; 0.10 and 0.5% mass) were added to separate CaCl_2_ and Na_2_SO_4_ aqueous solutions (0.2 mol·dm^−3^) and to deionized water. The solutions were then stirred using a magnetic stirrer for 15 min at 25°C. They were then separated from the aqueous phase (450 nm filter). The isolated CaCl_2_ and Na_2_SO_4_ aqueous solutions were analyzed for calcium and sulfate content and then diluted with deionized water, using shavings to create 0.1 mol·dm^−3^ stock solutions. The dilution compensated for any possible losses, such as those due to the sorption of some calcium and sulfate ions by wood. Subsequently, the stock solutions were mixed, and the conductivity of the resulting solution was measured in the identical manner as in the blank experiment.

**Figure 3:**
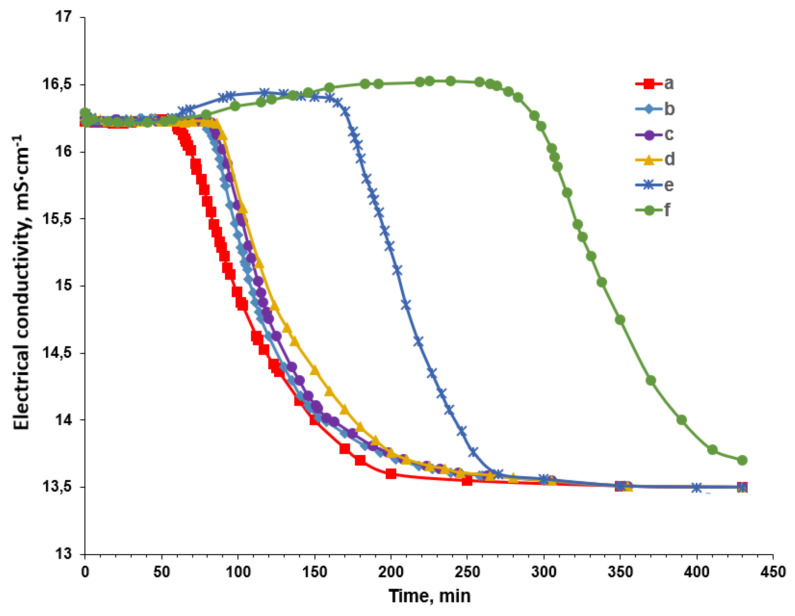
Conductivity as a function of time for 0.05 mg·dm^−3^ calcium sulfate solution at 25°C in a blank run (a), and in the experiments after treatment of the calcium and sulfate stock solutions with 0.05% (b), 0.1% (c), 0.5% (d) wood chips; in the presence of 0.1 mg·dm^−3^ of PAA-F1 (e), and in presence of 0.1 mg·dm^−3^ of PAA-F1 after treatment with wood chips, taken at 0.1% (f).

Finally, the sequential treatment of supersaturated solutions using spruce wood shavings and PAA-F1 was tested. For this purpose, both CaCl_2_ and Na_2_SO_4_ stock solutions were pre-treated with wood shavings. The shavings were then removed and the sulfate concentration was corrected and diluted with deionized water treated with wood chips to give a concentration of 0.1 mol·dm^−3^. The Na_2_SO_4_ solution was supplied with PAA-F1 solution and then mixed with calcium chloride solution in a volume ratio of 1:1. The results are shown in Figure 3 and Table 2. All experiments were performed in two replicates.

**Figure 4:**
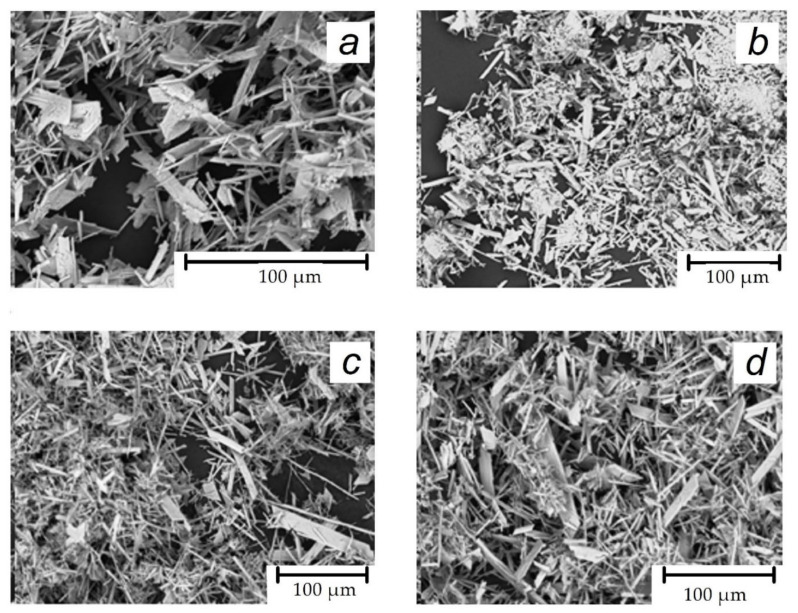
SEM images of gypsum crystals, deposited from the blank supersaturated solution (**a**), from the solution containing 0.1% wood-chip-treated ingredients (**b**), in the presence of 0.1 mg·dm^−3^ of PAA-F1 (**c**) and in the presence of 0.1 mg·dm^−3^ of PAA-F1 after 0.1% wood chip treatment (**d**).

After 24 h of interaction between CaCl_2_ and Na_2_SO_4_, the solid gypsum crystals were isolated, rinsed with water, dried at 50°C, and analyzed using SEM microscopy. The results are presented in Figure 4. 

### 2.3. Characterization of the Spruce Wood Shavings

As spruce wood chips are non-traditional materials in water treatment technologies, some additional features of their interactions with deionized water, calcium chloride and disodium sulphate solutions have been studied. These auxiliary experiments are intended to clarify whether some additional constituents are released from the wood into the deionized water and the stock solutions, and to assess the degree of calcium and sulphate sorption by the shavings. The procedure lasted 15 min with a magnet stirring at 300 rpm of 0.05, 0.10 and 0.50 mass % shavings in deionized water, calcium chloride and disodium sulfate solutions (0.1 mol·dm^−3^) at 25°C. The shavings were filtered out using a 450 nm filter prior to analyzing the liquid phase via ICP, chromatography, dynamic light scattering, and particle counter. The obtained results are presented in Table 3, Table 4, Table 5 and Table 6.

These data demonstrate a slight increase in conductivity for the deionized water due to some release of electrolytes from the chips (Table 6) and a change in pH from 5.9 to 6.1. At the same time, the shavings, taken in a quantity of 0.5 mass %, provide some sorption of calcium from a 0.1 mol·dm^−3^ CaCl_2_ solution (approximately 1%) and of sulfate anion from a 0.1 mol·dm^−3^ Na_2_SO_4_ stock solution (4.7%). Thus, an appropriate correction for sulphate stock solutions was made after their contact with shavings but before being mixed with the CaCl_2_ stock solution. The 1% variation in calcium concentration for the latter was deemed negligible; thus, no correction was carried out in this instance.

After 10 min of contact between 0.1% shavings and a 0.1 mg·dm^−3^ solution of PAA-F1, the fluorescence indicated a roughly 10% loss of antiscalant concentration due to sorption. As a result, the co-presence of shavings and PAA-F1 was deemed undesirable, and the sequential application of these materials was applied in the present study. 

### 2.4. Instruments 

The morphology of the deposited gypsum crystals was analyzed using a Hitachi TM3030 scanning electron microscope (SEM), Hitachi-High-technologies Corporation, Tokyo, Japan. SEM sample examinations were conducted using 15 kV voltage acceleration with a charge-up reduction mode. The crystal phase was located on a carbon conducting tape; the working distance was 4.1 mm. The SEM instrument was connected to an energy dispersive X-ray spectroscopy (EDS) analysis unit, Quantax 70, Hitachi-High-technologies Corporation, Tokyo, Japan, which was calibrated with a copper standard. Spot Mode was used for quantitative element analysis. 

The quantitative characterization of the suspended solid impurity concentration of the particles sized ≥ 100 nm was performed with an SLS-1100 particle counter (Particle Measuring Systems Inc., Boulder, CO, USA). As the concentration of particles in the unfiltered solutions was high, the corresponding stock solution probe was diluted 100-fold with deionized water, the measurement was performed, and the results were recalculated back to the initial concentration. For the gypsum solutions, direct experimental measurements were not carried out as the time taken for measuring was comparable with the nucleation period. Therefore, the concentration of nano/microparticles at the point of mixing calcium and sulfate brines was determined based on the mean value of the respective stock solutions.

The liquid phase was analyzed using dynamic light scattering (DLS) to qualitatively characterize impurities suspended as solid particles within a hydrodynamic diameter range of 1 nm to 1000 nm. DLS experiments were conducted at a temperature of 25°C using a Malvern Nano ZS instrument (Malvern Panalytical Ltd., Malvern, UK) with an operating power of 4 mW, wavelength λ = 633 nm, and scattering angle Θ = 173°. 

Specific conductivity was measured at 25°C with an Ekspert-002-2-6 conductivity meter (OOO Ekonics-Ekspert, Ekaterinburg, Russia) in a 100 mL thermostated glass beaker (100 mL), equipped with magnetic stirrer. The induction time was accurately determined by identifying the inflection point of the specific conductivity–time curve. 

The mass concentration of PAA-F1 in the test solutions was determined using spectrofluorimetric analysis (Shimadzu RF-6000 spectrofluorimeter, Shimadzu, Kyoto, Japan) in 10 mm cuvettes. The fluorescence intensity of the solution was registered at 460 nm, using an excitation wavelength of 375 nm. To establish the calibration curve for a mass concentration range of PAA-F1 between 0.5 and 15 mg·dm^−3^, a set of solutions with concentrations of 0.5, 1.0, 2.5, 5.0, 10.0 and 15.0 mg·dm^−3^ were prepared from the stock solution of PAA-F1. The solutions were analyzed immediately after preparation. The fluorescence intensity of the solution was measured at 460 nm. The calibration curve is plotted in the coordinates “fluorescence intensity—mass concentration of PAA-F1” (mg·dm^−3^). The slope is described by the function y = a·x. The calibration response is fitted by the least squares method.

## 3. Results

### 3.1. The Blank Experiment

The electrical conductivity of the control experiment varies over time (see (a) in Figure 3), in keeping with our previously published findings [23]. The induction time (55 min) is concordant with that reported in other studies on gypsum crystallization employing similar saturation index and temperature values [21,38,39]; refer to Table 7. It is likely that the discrepancy from reference [23] is attributable to variance in purity of calcium chloride, as the stock solution adopted in this study contained fewer impurities and particulate matter; refer to Table 2. The conductivity decreased steadily from 16.2 mS·cm^−1^ to 13.5 mS·cm^−1^ over the course of 150 min. 

Scale formation occurs in the bulk aqueous medium, resulting in the formation of typical gypsum crystals. The formation of these crystals can be observed in Figure 4a. According to [21], a supersaturation of SI ≤ 2.47 corresponds to heterogeneous nucleation at 30°C. Therefore, in our case, where SI = 2.3, the formation aligns well with the heterogeneous mechanism. It is important to note that both stock solutions, CaCl_2_ and Na_2_SO_4_, contain approximately 10^5^ solid particles larger than 100 nm in 1 mL. These are recorded by the particle counter. The corresponding data are shown in Table 1. Our previous research [16] has revealed the unavoidable presence of a smaller particle fraction (1 nm ≤ size ≤ 100 nm), with concentrations reaching at least 10^7^ units per 1 mL. Consequently, these nano/micro-impurities might act as crystal formation centers for gypsum. Element analysis of stock solutions indicates the potential presence of at least three chemical elements that could form solid impurities: aluminum, iron, and silicon. Table 5 provides the relevant numerical data. Silicon may exist in the form of quartz, calcium silicate, or aluminosilicate fine particles. Chemical speciation unequivocally indicates that iron and aluminum exclusively exist as solid species of Fe(OH)_3_ and Al(OH)_3_ in our solutions at a pH of 4–7. Therefore, the concept of heterogeneous nucleation of gypsum has a strong foundation.

### 3.2. Gypsum Crystallization from the Solution, Pretreated by the Spruce Wood Shavings 

In the sample pretreated with shavings, the conductivity gradually decreases from 16.2 mS·cm^−1^ to 13.5 mS·cm^−1^ over approximately 150 min with a slope similar to that of the blank experiment. This is illustrated in (b)–(d) in Figure 3. However, the small amount of spruce wood shavings ranging from 0.05 to 0.5% mass used to briefly treat the stock solutions revealed a sustainable and reliably recorded effect of increasing the induction time from 55 min to 85 min. These changes are shown in Figure 3 and Table 2. This effect cannot be attributed to the reduction of SI through the sorption of calcium and sulfate ions on the surface of the shavings. According to Table 4, there have been no changes in the concentrations of either [Ca^2+^] or [SO_4_^2−^]. The observed delay in gypsum fouling is unlikely to be caused by emissions of organic wood ingredients that may possess antiscalant properties. This is due to the absence of such ingredients detected by ion chromatography or gas chromatography. The former technique only identified the release of calcium and sulfate ions into the water. Relevant data can be found in Table 6. Similarly, Table 5 shows that wood shavings have emitted some species of Mg, Si, and Cu into calcium and sulfate solutions. This has been detected through ICP analysis. However, it should be noted that Fe and Al have been absorbed from the stock solutions. The initial iron content in the blank experiment decreased from 0.258 mmol·dm^−3^ to 0.178 mmol·dm^−3^, indicating a 31% loss in the shavings treated gypsum solution. Similarly, the aluminum concentration decreased from 1.6 mmol·dm^−3^ to 1.18 mmol·dm^−3^, indicating a 26% loss. Taking into account that these two elements have to be completely hydrolyzed under experimental conditions, it is probable that these impurities are present in the form of colloidal nanoparticles: Fe(OH)_3_^solid^ and Al(OH)_3_^solid^. As far as Fe(OH)_3_^solid^ nanoimpurities are known to disable polyacrylate as a scale inhibitor and to promote gypsum scale formation [24], it is reasonable to suppose that the gypsum nucleation kinetic in the blank experiment is largely determined by these specific nanoimpurities. Thus, the wood shavings bind Fe(OH)_3_^solid^ and Al(OH)_3_^solid^ nanoimpurities, reducing the number of gypsum nucleation centers and consequently increasing the induction time of CaSO_4_·2H_2_O crystal formation. Notably, Figure 4b illustrates no crystal habit change or modification. 

Simultaneously, the majority of the particulate matter with a particle size greater than 100 nm in the shaving-treated samples (Table 3) remains unaffected when compared to untreated samples (Table 1). Furthermore, it appears that the wood releases some additional fine solids (provisionally identified as silica), as evidenced by a notable shift in the dominant species from approximately 300 nm (Table 1) to roughly 1 nm (Table 3) detected using DLS. Evidently, not all solid nano/micro-impurities are capable of acting as nucleation agents for gypsum formation. The data suggest that there is a correlation between the decrease in Al and Fe content and an increase in induction time. This leads to the assumption that Fe(OH)_3_^solid^ and Al(OH)_3_^solid^ solids play a significant role in the nucleation process. Additionally, it has been found that spruce wood shavings act as selective traps for these solid impurities.

### 3.3. Gypsum Crystallization in the Presence of PAA-F1 and after Sequential Treatment with Wood Shavings/PAA-F1 

In the presence of PAA-F1, the conductivity shows a slight increase (see (e) in Figure 3) presumably due to the interaction of the polyacrylate with calcium ions and a corresponding release of some protons from the polymer. Subsequently, the conductivity decreases from 16.5 mS·cm^−1^ to approximately 13.5 mS·cm^−1^ in about 150 min. Interestingly, the presence of PAA-F1 ((e) in Figure 3) does not appear to have an impact on the crystal growth steps, as evidenced by the fact that the electrical conductivity drop has the same slope and duration as the blank run ((a) in Figure 3). However, the induction time between runs is significantly different, with a significant increase from 55 min in the blank run to 165 min in the presence of 0.1 mg·dm^−3^ PAA-F1. This finding aligns with the well-established effectiveness of polyacrylates in preventing gypsum scale formation [25,37]. It is worth noting that the deposited CaSO_4_·2H_2_O crystals exhibit the same characteristics as those in the control group, as evident from Figure 4c. Therefore, PAA-F1’s primary role is in facilitating the nucleation stage, rather than influencing any subsequent crystal growth, aggregation, or sedimentation processes.

The most remarkable results were obtained after the sequential treatment with wood shavings/PAA-F1. The initial solutions were subjected to treatment with wood shavings and subsequently filtered. Na_2_SO_4_ solution was then treated with PAA-F1 and allowed to reach equilibrium before being merged with CaCl_2_ solution in a 1:1 ratio. The conductivity showed a slight increase, as observed in the case of PAA-F1 in untreated shavings. However, there was a significant decline in conductivity due to the formation of the solid phase of gypsum. The curve shape ((f) in Figure 3) was similar to all the other cases ((a)–(e) in Figure 3), but the maximum value of induction time reached 260 min. Notably, the study highlights a synergistic effect between sequential 0.1% wood mass savings and PAA-F1 treatment. Indeed, Δt_ind_ = t_ind_ − t_ind_^blank^ comprised 205 min, while for the sample treated with shavings and for the experiment with PAA-F1, it composed only 25 and 110 min, respectively. The relevant data are detailed within Table 2. Therefore, a promising approach for water treatment appears to be the combined usage of spruce wood shavings followed by polyacrylate injection. Wood shavings are a cost-effective and ecologically sustainable raw material [40], which can be removed from treated water using coarse water filtration. However, significant research is still required to determine the most suitable wood, size and roughness of the shavings, and the optimal conditions for using this fibrous material in water pretreatment.

## 4. Discussion

Present research provides further evidence of the significant influence of naturally occurring solid nanoimpurities in the formation and inhibition of inorganic scale [21,23,24]. It is widely accepted that the nucleation of sparingly soluble salts in the bulk occurs heterogeneously at low SI values, with natural nano/microimpurities acting as nucleation centers [17,18,21]. However, many studies on antiscalants attribute the scale inhibition effect to either inhibitor sorption onto the pre-existing scale surface or to the highly uncertain mechanism of antiscalant impact on homogeneous nucleation [1,7,25,26,27,29,32]. Meanwhile, our group’s application of fluorescent-tagged antiscalants revealed that the scale inhibitor predominantly obstructs the surfaces of nano/micro-impurities rather than CaSO_4_·2H_2_O embryos [30,31].

Actually, this statement is drawn from indirect evidence, as modern research methods do not offer any reliable tools for the quantitative characterization of natural particulate matter with sizes between 1 and 30 nm. However, in shavings-treated stock solutions, the particles with a hydrodynamic diameter of roughly 1 nm dominate, and they can be detected by DLS in untreated solutions. Therefore, our interpretation is based on indirect evidence. We posit that the wood shavings selectively absorb the Fe(OH)_3_^solid^ and Al(OH)_3_^solid^ nanoimpurities, which are known to render polyacrylates ineffective as antiscalants during gypsum scale formation [24], and to promote scale nucleation [41]. Thus, the quantity of possible nucleation centers decreases, leading to an extension in the induction period. Polyacrylate lengthens the induction period by obstructing the surface of nano-sized impurities on Fe(OH)_3_^solid^ and Al(OH)_3_^solid^ in a somewhat dissimilar manner. The use of wood shavings in combination with PAA-F1 produces a synergistic effect, as shown in Table 2. It is evident that the shavings promote the action of the inhibitor by consuming a significant proportion of the nucleation centers.

However, it is important to note that not all types of nanoparticles equally promote crystallization. The shavings result in a higher relative concentration of particles with a size of approximately 1 nm. This conclusion is drawn from a comparison of DLS data presented in Table 1 and Table 3. Tentatively, it appears that this is due to the release of some silica nanoparticles from the wood into the stock solutions, as indicated in Table 5 and Table 6. Interestingly, the activity of silica nanoparticles seems to be considerably less than that of iron and aluminum. Undoubtedly, colloidal SiO_2_ deactivates PAA much less than solid Fe(OH)_3_^solid^ [24]. In general, the finest fraction of particulate matter continues to pose a challenge for research in water treatment. This area is largely ‘terra incognita’ for the water treatment community. Recent regulations [42,43,44,45] have not yet standardized it, although they mainly address fractions with a particle size over 1 µm. Even for ultra-pure water used in the semiconductor industry [46], the most stringent standard only regulates particles with a size of ≥50 nm. Therefore, little is currently known about the specific chemical composition of particulate matter. It is tentatively suggested that the structure is a mixture of particles derived from various sources and is heavily influenced by its chemical composition. It is important to note that a blend of fine colloids in a gypsum solution, which has been artificially prepared using reagent grade chemicals, could differ from one obtained from natural municipal water through its saturation. Therefore, the effectiveness of the antiscalant is likely to be different. We emphasize that our analyses cannot be currently refuted or confirmed experimentally due to the absence of adequate research methods. However, we aim to present alternative explanations for experimental observations for researchers. Nevertheless, the documented collaboration of feed water pretreatment utilizing wood shavings, succeeded by polyacrylate injection, appears to be a significant discovery for water treatment technologies that extend beyond gypsum scale development.

## 5. Conclusions

Wood shavings have the ability to slow gypsum nucleation in supersaturated solution that is not directly present in the reaction vessel. The addition of spruce wood shavings (0.1% by mass) to CaCl_2_ and Na_2_SO_4_ stock solutions has been shown to prolong the induction time t_ind_ in 0.05 mol·dm^−3^ freshly prepared gypsum solutions (saturation SI = 2.3) by 25 min at ambient temperature compared to the blank run (t_ind_^blank^). This phenomenon is dependent on the quantity of shavings and is concomitant with the release of certain elements (Mg, Cu, P, Si) from the wood into the aqueous phase, and the absorption of Al and Fe from stock solutions.

The shavings indicate an ability to enhance the efficacy of the antiscalant polyacrylate PAA-F1. In the presence of conventional PAA-F1 (0.1 mg·dm^−3^), the difference Δt_ind_ = t_ind_ − t_ind_^blank^ is 110 min, while using shavings to treat stock solutions followed by PAA-F1 injection results in Δt_ind_ = 205 min. 

The observed synergism is attributed to the selective elimination of colloidal Fe(OH)_3_^solid^ and Al(OH)_3_^solid^ nanoimpurities from the CaCl_2_ and Na_2_SO_4_ stock solutions through their sorption onto the well-developed surface of the wood. This makes wood shavings a very promising, cheap, renewable and environmentally friendly material that can significantly improve the effectiveness of any antiscalant. However, it is currently difficult to estimate the economic feasibility of this option since the spruce wood shavings may not be optimal. According to the Russian domestic market, the price of 1 kG of PAA (100%) is around USD 4, while shavings for pets cost around USD 0.6. However, it is important to test shavings that are ten times cheaper before making a definite statement. Additionally, the efficacy of shavings in the flow of feeding water and their ability to regenerate and be reused is currently unclear. Therefore, further research is necessary to determine the ideal shavings material and optimal conditions for its use in water treatment. 

## Figures and Tables

**Table 1:** Solid impurities in 0.1 mol·dm^−3^ CaCl_2_ and Na_2_SO_4_ solutions and in deionized water, detected using a particle counter and DLS.

Sample	pH	DLS Particle Distribution by Number, nm	Cumulative Natural Solid Impurity Concentration, Measured Using a Particle Counter, Number of Particles in 1 mL			Ref.
			>100 nm	>200 nm	>300 nm	
Deionized water	5.66	-	225 ± 23	28 ± 5	11 ± 4	Present work
	6.3	-	320 ± 10	60 ± 10	23 ± 8	[23]
CaCl_2_ solution	6.7	440 ± 200 (100%)	(380 ± 60) × 10^3^	(48 ± 3) × 10^3^	(15 ± 1) × 10^3^	Present work
	9.8	c.a. 1.0	(480 ±70) × 10^3^	(180 ±40) × 10^3^	(80 ± 20) × 10^3^	[23]
Na_2_SO_4_ solution	6.3	280 ± 150 (100%)	(390 ± 50) × 10^3^	(86 ± 4) × 10^3^	(34 ± 3) × 10^3^	Present work
	6.7	c.a. 1.0	(370 ± 60) × 10^3^	(90 ± 20) × 10^3^	(30 ± 6) × 10^3^	[23]

**Table 2:** Impact of treatment of stock solutions on induction time of gypsum scale formation at (25 ± 1) °C for SI = 2.3.

Treatment Mode		Induction Time, min	Δt_ind_ = t_ind_ − t_ind_^blank^, min
Shavings, % Mass.	PAA-F1, mg·dm^−3^		
0	0	55 ± 5	0
0.05	0	75 ± 5	20
0.1	0	80 ± 5	25
0.5	0	85 ± 5	30
0	0.1	165 ± 5	110
0.1	0.1	260 ± 5	205

**Table 3:** Solid impurities in 0.1 mol·dm^−^^3^ CaCl_2_ and Na_2_SO_4_ solutions and in deionized water, detected using a particle counter and DLS after 15 min contact with wood shavings at 25°C.

Sample	Wood Shavings Mass, %	DLS Particle Distribution by Number, nm	Cumulative Natural Solid Impurity Concentration, Measured Using a Particle Counter, Number of Particles in 1 mL		
			>100 nm	>200 nm	>300 nm
Deionized water	0.1	-	225 ± 23	28 ± 5	11 ± 4
CaCl_2_ solution	0.05	0.7 ± 0.1 (100%)	(380 ± 50)·10^3^	(40 ± 2)·10^3^	(12 ± 1)·10^3^
	0.10	0.7 ± 0.1 (100%)	(420 ± 40)·10^3^	(87 ± 8)·10^3^	(20 ± 5)·10^3^
	0.50	0.65 ± 0.09 (100%)	(345 ± 60)·10^3^	(90 ± 3)·10^3^	(17 ± 4)·10^3^
Na_2_SO_4_ solution	0.05	0.7 ± 0.1 (100%	(407 ± 40)·10^3^	(54 ± 7)·10^3^	(24 ± 9)·10^3^
	0.10	0.7 ± 0.1 (100%)	(395 ± 20)·10^3^	(70 ± 8)·10^3^	(17 ± 5)·10^3^
	0.50	1.8 ± 0.5 (100%)	(410 ± 30)·10^3^	(62 ± 9)·10^3^	(18 ± 4)·10^3^

**Table 4:** The effect of wood shavings mass on 0.1 mol·dm^−3^ CaCl_2_ and Na_2_SO_4_ solutions after 15 min contact at 25°C.

Parameter	0.1 mol·dm^−3^ CaCl_2_ Solution				0.1 mol·dm^−3^ Na_2_SO_4_ Solution			
Wood shavings concentration, % mass	0	0.05	0.10	0.50	0	0.05	0.10	0.50
pH	6.5	6.28	5.98	5.42	6.17	5.64	5.27	4.83
Conductivity, 25°C; mS·cm^−1^	18.36	18.24	18.21	18.19	16.77	16.76	16.78	16.5
Ca^2+^, mol·dm^−3^	0.1003 ^1^	0.1001 ^1^	0.1001 ^1^	0.0992 ^1^	-	-	-	-
SO_4_^2−^, mol·dm^−3^	-	-	-	-	0.1051 ^2^	0.1014 ^2^	0.1003 ^2^	0.0867 ^2^

^1^ Complexonometric titration with EDTA; ^2^ Complexonometric back-titration following sulfate precipitation with barium.

**Table 5:** The effect of wood shavings (0.1% by mass) on chemical element impurities in 0.1 mol·dm^−3^ CaCl_2_ and Na_2_SO_4_ solutions after 15 min contact at 25°C.

Parameter	0.1 mol·dm^−3^ CaCl_2_ Solution		0.1 mol·dm^−3^ Na_2_SO_4_ Solution	
	Initial	After Contact with Shavings	Initial	After Contact with Shavings
Ca, mg·dm^−3^	-	-	0.2065	5.2370
Mg, mg·dm^−3^	0.7876	1.3840	0.4987	1.1090
Fe, mg·dm^−3^	0.0001	0.0013	0.0288	0.0186
Al, mg·dm^−3^	0.0054	0.0004	0.0810	0.0634
Si, mg·dm^−3^	0.1522	0.3524	0.2247	0.2669
S, mg·dm^−3^	2.2380	2.7350	-	-
P, mg·dm^−3^	0.0424	0.0448	0.0314	0.0354
Cu, mg·dm^−3^	0.0616	0.9569	0.0449	0.0474
Na, mg·dm^−3^	18.870	18.810	-	-
Organic impurities ^1^	Not detected	Not detected	Not detected	Not detected

^1^ Ion and gas chromatography.

**Table 6:** Alternations in deionized water following a 15 min interaction with 0.5% mass of wood shavings at 25°C.

Parameter	Initial In-House Deionized Water	The Water after Contact with Shavings	Comment
Conductivity, 25 °C; µS/cm	0.25	23.2	
pH	5.90	6.12	
Ca, mg·dm^−3^	0.0000	41.070	ICP analysis
Mg, mg·dm^−3^	0.0000	29.740	
Fe, mg·dm^−3^	0.0002	0.0034	
Al, mg·dm^−3^	0.0000	0.0025	
Si, mg·dm^−3^	0.0000	7.2360	
S, mg·dm^−3^	0.0086	26.230	
P, mg·dm^−3^	0.0074	0.0884	
Cu, mg·dm^−3^	0.0058	0.0042	
Na, mg·dm^−3^	0.0000	13.650	
Organic impurities	Not detected	Not detected	Gas and ion chromatography

**Table 7:** Induction times of gypsum deposition for SI ranging from 2.2 to 2.7 and temperatures around 25 ± 5 °C.

Sat. Level	T,°C	Location of Nucleation Process	Experimental Conditions	Method of t_ind_ Measurement	t_ind_, min	Ref.
2.2	23	Bulk solution	Stirring of a supersaturated solution with a 3 cm octagonal magnetic stir bar coated with Teflon, at a rate of 200 rpm, in a polypropylene beaker.	Light transmittance decrease	63	[21]
2.6	30	Bulk solution	Most of details are not listed	Ca-ISE electrode	47	[38]
2.17	30	Bulk solution	pH 7.0; no removal of particulate matter in stock solutions is specified	conductivity	23	[39]
2.3	25	Bulk solution	Stirring of a supersaturated solution is achieved by using a polymer-coated magnetic stir bar at a rate of 700 rpm within a glass cell. No filtration of stock solutions is employed.	conductivity	8	[23]
2.3	25	Bulk solution	Stirring of a supersaturated solution by utilizing a polymer-coated magnetic stir bar rotating at a speed of 700 rpm within a glass container; filtering the stock solutions with a 200 nm membrane.	conductivity	16	[23]
2.3	25	Bulk solution	pH 6.0–7.2; stirring of a supersaturated solution using polymer-coated magnetic stir bar at rate 300 rpm in a glass cell	conductivity	55	Present work

## Data Availability

http://www.nc-mtc.ru/publikacii-2023-goda, accessed on 10 April 2023.
